# Current trend and development in bioinformatics research

**DOI:** 10.1186/s12859-020-03874-y

**Published:** 2020-12-03

**Authors:** Yuanyuan Fu, Zhougui Ling, Hamid Arabnia, Youping Deng

**Affiliations:** 1 Department of Quantitative Health Sciences, John A. Burns School of Medicine, University of Hawaii at Manoa, Honolulu, HI 96813 USA; 2 Department of Pulmonary and Critical Care Medicine, The Fourth Affiliated Hospital of Guangxi Medical University, Liuzhou, 545005 China; 3 Department of Computer Science, University of Georgia, Athens, GA 30602 USA

**Keywords:** Bioinformatics, Biomarkers, Human disease, Microbiome

## Abstract

This is an editorial report of the supplements to BMC Bioinformatics that includes 6 papers selected from the BIOCOMP’19—The 2019 International Conference on Bioinformatics and Computational Biology. These articles reflect current trend and development in bioinformatics research.

The supplement to BMC Bioinformatics was proposed to launch during the BIOCOMP’19—The 2019 International Conference on Bioinformatics and Computational Biology held from July 29 to August 01, 2019 in Las Vegas, Nevada. In this congress, a variety of research areas was discussed, including bioinformatics which was one of the major focuses due to the rapid development and requirement of using bioinformatics approaches in biological data analysis, especially for omics large datasets. Here, six manuscripts were selected after strict peer review, providing an overview of the bioinformatics research trend and its application for interdisciplinary collaboration.

Cancer is one of the leading causes of morbidity and mortality worldwide. There exists an urgent need to identify new biomarkers or signatures for early detection and prognosis. Mona et al. identified biomarker genes from functional network based on the 407 differential expressed genes between lung cancer and healthy populations from a public Gene Expression Omnibus dataset. The lower expression of sixteen gene signature is associated with favorable lung cancer survival, DNA repair, and cell regulation [1]. A new class of biomarkers such as alternative splicing variants (ASV) have been studied in recent years. Various platforms and methods, for example, Affymetrix Exon-Exon Junction Array, RNA-seq, and liquid chromatography tandem mass spectrometry (LC–MS/MS), have been developed to explore the role of ASV in human disease. Zhang et al. have developed a bioinformatics workflow to combine LC–MS/MS with RNA-seq which provide new opportunities in biomarker discovery. In their study, they identified twenty-six alternative splicing biomarker peptides with one single intron event and one exon skipping event; further pathways indicated the 26 peptides may be involved in cancer, signaling, metabolism, regulation, immune system and hemostasis pathways which validated by the RNA-seq analysis [2].

Proteins serve crucial functions in essentially all biological processes and the function directly depends on their three-dimensional structures. Traditional approaches to elucidation of protein structures by NMR spectroscopy are time consuming and expensive, however, the faster and more cost-effective methods are critical in the development of personalized medicine. Cole et al. improved the REDRAFT software package in the important areas of usability, accessibility, and the core methodology which resulted in the ability to fold proteins [3].

The human microbiome is the aggregation of microorganisms that reside on or within human bodies. Rebecca et al. discussed the tissue-associated microbial detection in cancer using next generation sequencing (NGS). Various computational frameworks could shed light on the role of microbiota in cancer pathogenesis [4]. How to analyze the human microbiome data efficiently is a huge challenge. Zhang et al. developed a nonparametric test based on inter-point distance to evaluate statistical significance from a Bayesian point of view. The proposed test is more efficient and sensitive to the compositional difference compared with the traditional mean-based method [5].

Human disease is also considered as the cause of the interaction between genetic and environmental factors. In the last decades, there was a growing interest in the effect of metal toxicity on human health. Evaluating the toxicity of chemical mixture and their possible mechanism of action is still a challenge for humans and other organisms, as traditional methods are very time consuming, inefficient, and expensive, so a limited number of chemicals can be tested. In order to develop efficient and accurate predictive models, Yu et al. compared the results among a classification algorithm and identified 15 gene biomarkers with 100% accuracy for metal toxicant using a microarray classifier analysis [6].

Currently, there is a growing need to convert biological data into knowledge through a bioinformatics approach. We hope these articles can provide up-to-date information of research development and trend in bioinformatics field.

## Data Availability

Not applicable.

## Abbreviations

BIOCOMP’19: The 2019 International Conference on Bioinformatics and Computational Biology
LC–MS/MS: Liquid chromatography tandem mass spectrometry
ASV: Alternative splicing variants
NMR: Nuclear Magnetic Resonance
REDCRAFT: Residual Dipolar Coupling based Residue Assembly and Filter Tool
NGS: Next generation sequencing
